# Tinnitus Measured in Everyday Life: A Literature Review of Ecological Momentary Assessment Studies

**DOI:** 10.1007/s10162-025-00995-0

**Published:** 2025-06-09

**Authors:** Milena Engelke, Sebastian Müller, Berthold Langguth, Rüdiger Pryss, Winfried Schlee

**Affiliations:** 1 Department of Psychiatry and Psychotherapy, University of Regensburg, Regensburg, Germany; 2 Institute for Information and Process Management, Eastern Switzerland University of Applied Sciences, St. Gallen, Switzerland; 3 Institute of Clinical Epidemiology and Biometry, University of Würzburg, Würzburg, Germany

**Keywords:** Tinnitus, Ecological momentary assessment, Experience sampling, Longitudinal data, Digital health, Personalised medicine

## Abstract

Tinnitus, a common auditory phenomenon, often presents with considerable between-person heterogeneity and within-person fluctuations. To understand the pathophysiological mechanisms and advance patient-centred care, it is essential to recognise these variations. Ecological Momentary Assessment (EMA) is a (close-to) real-time data collection method that offers insights into short- and long-term fluctuations of subjective symptoms and their interaction with psychological, environmental, and physiological factors. EMA applied in tinnitus research has shown promise in capturing the nuances of tinnitus experience in naturalistic settings, minimizing recall bias inherent in traditional retrospective methods. This narrative literature review aims to provide a comprehensive up-to-date picture of EMA in tinnitus research by describing previous and current applications, summarising scientific findings, and identifying research gaps by drawing lessons from adjacent mental health fields. 28 publications were identified and assigned to six different topics based on thematic and methodological matters. We highlight contributions of EMA methodology for tinnitus research such as findings on momentary and longitudinal symptom interactions, circadian rhythms, individual differences in symptom patterns and its contributions to treatment evaluation. Emerging technologies, including machine learning, are opening new avenues for personalised tinnitus understanding and management. Despite promising advances, challenges such as data reliability, participant compliance, and integration with sensor-based passive data collection remain areas for further exploration. Drawing lessons from adjacent mental health fields, we propose future directions for EMA in tinnitus research, emphasizing the integration of multimodal data, advanced analytics, and ecological validity to enhance the understanding and management of chronic tinnitus.

## Introduction

In this paper, we provide a literature review on the use of Ecological Momentary Assessment (EMA) in tinnitus research. We begin by introducing the concept of EMA, followed by an overview of tinnitus as a clinical condition.

### Ecological Momentary Assessment

The momentary assessment of human behaviour and emotion outside the laboratories has a long history. One of the first pioneers was J.C. Flügel, who was interested in the ratio of positive and negative experience in human life in the 1920 s [1]. To study this, retrospective evaluation was clearly rejected because, in his view, “even to emotionally stable individuals the value of life as measured in terms of pleasure and pain will appear to vary very considerably according to the dominant feeling tone of the passing moment”. The logical conclusion to apply very frequent assessments was likewise discarded due to the immense irritability which could potentially influence the observed phenomenon. Instead, he conducted assessments at varying intervals that were defined by the natural course of the day. Participants were asked to frequently keep record of the pleasant and unpleasant feelings and their accompanying mental state in their daily life, ideally every hour, over a period of 30 days. Comparing the odd with the even days, the author found satisfactory reliability estimates for all except the extreme values of pleasure and unpleasure. Overall, the nine studied participants experienced more pleasure than unpleasure in their daily life with considerable between-subject differences.

In 1977, Csikszentmihalyi and colleagues were interested in the interaction of adolescents’ daily behaviour and feelings [2]. Similar to Flügel, they were convinced that “in order to understand adolescents one needs to know what they do all day and the pattern of feelings associated with what they are doing” which resulted in the need for a “systemic, ecological approach”. They randomly sampled self-reports of adolescents’ activities and emotions during a normal week. The subjects were prompted by audio signals through an electronic paging device 5–7 times per day to fill out a paper–pencil form with questions about their current location, activity, rationale for the activity, environmental interaction, mood and physical state. The authors found that adolescents were primarily engaged in talking to peers and watching television, while among their rarest activities were studying or other kind of work. Peer communication was mostly rated as voluntary activity and associated with happy mood, while watching television was motivated by the lack of alternatives and associated with the absence of any feelings.

This was one of the first studies using the Experience Sampling Method (ESM) which was further developed by Csikszentmihalyi and colleagues in the following years [3]. It is characterized by individuals reporting their cognitions, emotions, and behavior in real-time in response to random prompts throughout the day. This methodological approach laid the groundwork for Csikszentmihalyi’s well-known research on the flow concept. With data gathered from individuals as they went through their daily activities, he found out that engaging in intrinsically motivated activities that were optimally balanced between being challenging and matching participants’ skill level increased the probability of experiencing flow, a condition of total immersion in an activity [4].

The methodology of repeated sampling was refined in the pioneering work of Stone and Shiffman in the 1990 s. They established the concept of Ecological Momentary Assessment (EMA), which they defined as “monitoring or sampling strategies to assess phenomena at the moment they occur in natural settings; thus maximizing ecological validity while avoiding retrospective recall” [5]. Thus, they extended the ESM methodology by allowing for different sampling schedules next to random prompts. Also, while ESM was hitherto used in psychological research to study daily life experiences, mental states, and the dynamics of subjective well-being, Stone and Shiffman adapted it to the clinical setting to study health-related behaviors, experiences, and symptoms [6]. Today, ESM and EMA are mostly used synonymously.

EMA is intended to reduce potential recall bias by assessing phenomena from the current moment, requires careful scheduling of records depending on the subject of interest, includes repeated assessment of the same individual and emphasizes ecological validity by monitoring in the natural environment. Stone and Shiffman themselves routed EMA not only in the tradition of experience sampling, but also in the tradition of naturalistic observation [5]. Naturalistic observation involves the direct, unobtrusive observation of behaviour in its most natural form, mostly by trained observers who collect the monitored events by means of a coding system [7].

EMA has evolved to a methodological modular system with varying assessment schedules, technologies in use, subjects and topics under surveillance, yet, with the common ground of repeated, (close to) real-time data collection in the subjects’ natural environment [8]. It is now frequently used in medical research to study the natural fluctuation of clinical symptoms or phenomena that elude objective measurement, such as tinnitus.

Despite its rapid spread, it should be noted that EMA, in its current form, requires some preconditions such as vision, literacy, the ability to reflect on its own condition, the knowledge of operating a smartphone, Internet connection (at least from time to time) as well as perseverance to answer the questions over a certain period. This makes it inappropriate in studying certain populations such as infants, persons suffering some forms of dementia or with certain physical handicaps as well as studying under certain conditions such as in very remote regions.

### Tinnitus as a Clinical Condition

Tinnitus refers to the perception of sound in the absence of acoustic stimulation which is often caused by abnormalities along the auditory pathway [9]. Such abnormalities, including damaged hair cells, can result from factors like aging, sensorineural hearing loss and ear infections [10]. In some instances, tinnitus may resolve if the underlying cause, such as otitis media, is treated. Yet, in some cases, it becomes chronic as a consequence of neural changes in the central auditory system [9]. The reasons for manifestation are still largely unknown, but psychological mechanisms might play a role [11]. Chronic tinnitus, which is defined persisting for at least 6 months, affects around 10% of the whole population [12]. Approximately 2% experience severe tinnitus which is defined as being bothered by the tinnitus perception [12] and can occur comorbidly with other symptoms, such as difficulties concentrating, sleep disturbances, reduced sound tolerance, persistent irritation [13]. There is a high comorbidity with mental health diseases such as anxiety and depression with probably bidirectional causality, such as tinnitus patients developing depression and depressed patients being at increased risk for developing tinnitus [14]. A major challenge for researchers and clinicians is the heterogeneity of tinnitus, both in its perceptual characteristics and its underlying mechanisms, as well as its impact on individuals’ lives [15].

Given these complexities, it becomes evident that understanding the daily fluctuations and the heterogeneity in real-time experiences of individuals with tinnitus is crucial. This is where EMA emerges as a valuable tool, offering a method to capture longitudinal data in real-time from patients in their natural environments, thereby providing insights that are difficult to obtain through established methods of health status recording.

## Methods

This narrative literature review aims to provide a comprehensive up-to-date picture of EMA in tinnitus research by describing previous and current applications, summarising scientific findings, and identifying research gaps by drawing lessons from adjacent mental health fields. A non-systematic search has been performed in PubMed using the terms “tinnitus” and “ecological momentary assessment” or “experience sampling”. Original research studies which mainly investigated tinnitus with EMA methodologies and references cited therein were included. The last search was conducted in January 2025.

28 publications were included and assigned to six different topics that were identified based on thematic and methodological matters. In Chapter 1 of the Results section, findings from non-systematically selected EMA studies from adjacent mental health fields are described which provide a basis to identify research gaps and propose future directions for EMA in tinnitus research. In Chapter 2, we describe the evaluation of the technical implementation and compare study methods of the included publications. Scientific findings are summarized according to the identified topics in Chapter 3.

## Results

### Chapter 1: Ecological Momentary Assessment in Mental Health Research

In this chapter, we critically examine EMA research findings from adjacent fields, such as well-being, mental health, mood and psychotic disorders, to gain a better understanding of how this methodology fills in research gaps. Further, we address both strengths and limitations of the methodology which must be considered, especially when adapting this approach to tinnitus research. The compilation is far from exhaustive, but should provide insights into the potentialities and challenges of EMA research.

#### Fine-Grained Evaluation of Fluctuations

The EMA methodology enables repeated sampling within-day and over longer time periods that allows to study both short-term as well as long-term patterns of self-reported symptoms. Stieger and Reips investigated daily and weekly fluctuations of well-being with three daily assessments over a two-week period in 213 participants and found that individuals felt better in the evening and on the weekends [16]. An earlier hypothesized “blue Sunday effect”, i.e. individuals feeling worse on Sundays, could be refined by a more detailed resolution of measurement times. Sunday ratings from the morning until noon followed the pattern of increasing mood of the other days. The difference started to become apparent in the afternoon: While mood increased further during afternoon on Mondays to Saturdays (M_evening_ = 4.53), mood level on Sundays stagnated (M_evening_ = 0.31; *t* = 4.10, *p* < 0.001, *d* = 0.23). Thus, not the whole Sunday, but only the second half might be “blue” [16].

Houben et al. conducted a meta-analysis to study the association between emotional dynamics and well-being measured with EMA across the time scales of seconds, hours, or days (max. one week) [17]. Results showed a negative correlation of well-being with emotional variability (i.e. range of emotional states across time, *ρ* = −0.178, *p* = < 0.001), emotional instability (i.e. magnitude of emotional changes from one moment to the next, *ρ* = −0.205, *p* = < 0.001), and emotional inertia (i.e. ability to predict emotional state from the previous moment, *ρ* = −0.151, *p* = < 0.001). It was concluded that people high in well-being exhibit a moderate emotional reactivity as well as an intact emotional regulation to events resulting in smaller deviations from the baseline level [17].

While these findings highlight EMA's strength in capturing subtle temporal patterns, they rely on frequent engagement from participants over days or weeks. It is suspected that participant fatigue can impact both compliance and data quality, particularly in clinical populations. Meta-analytical evidence estimates average compliance to be around 80%, with no differences between healthy and clinical populations [18–21]. Only psychotic patients were found to be less compliant than healthy participants [19]. However, the impact on data quality remains difficult to assess, and compliance rates may also be affected by self-selection bias (inclusion of only EMA-motivated participants).

#### High Ecological Validity of Measurement

EMA is characterized by high ecological validity catching the participant within his or her ordinary daily life. Findings known from retrospective evaluation can be replicated or refined by EMA data as well as supported with objective measures. MacKerron and Mourato performed a large study with more than 20.000 participants to investigate the relationship between self-rated momentary well-being and immediate environment obtained from GPS data of more than one million data points [22]. Controlling for potential confounders such as weather, daylight or activity as well as subject-level differences including demographic information, they found that feeling better was related to being in natural surroundings. Some confounders were identified through open-source data (e.g. weather) and were matched with the exact location and timestamp, others were measured with EMA (e.g. activity) [22].

A similar investigation that studied associations between self-rated momentary well-being and self-rated surroundings extended this finding by time-lasting effects of natural surroundings on happiness [23]. Seeing trees and the sky predicted well-being at the next assessment which appeared to be on average 2.5 h later. The feeling of being in contact with nature predicted well-being on average 5 h later in the second next measurement [23].

Myin-Germeys et al. investigated emotional reactivity to daily life stress in psychotic, depressed and bipolar patients as well as healthy controls [24]. Vulnerability to extreme life events in these psychopathological disorders is well-known from retrospective research, the authors aimed to extend this finding to daily stress reactivity using EMA methodology. Depressed and psychotic patients exhibited a higher daily stress reactivity in negative affect compared to bipolar patients and healthy controls, while psychotic and bipolar patients showed a higher daily stress reactivity in positive affect compared to depressed patients and healthy controls. Those differences were only apparent for activity-related stress but not for social stress [24]. Thus, vulnerability to stress for severe mental illnesses could be extended to more subtle daily life stressors and re-fined for different diagnoses, types of stress and affective states.

The same group followed schizophrenic patients and healthy controls with EMA over six consecutive days in another study [25]. They aimed to investigate momentary emotional experiences and hedonic capacities of this group of patients since they are often said to have deficits in emotion expression and hedonic experiences. Patients exhibited higher intensity in negative affect and lower intensity in positive affect compared to controls. Also, patients reported experiencing fewer positive events. However, when experiencing a positive event, hedonic experience was equally strong for patients and controls (χ^2^(3) = 1.69, *p* = 0.64). The same effect was found for social hedonic capacity: Even though patients were less often in company of others, they expressed the same level of positive affect when in company with others compared to controls (β = 0.01, 95% CI = 0.08–0.06, *p* = 0.76). Thus, there was no empirical evidence for specific deficits in emotional experiences nor hedonic capacities. The difference appeared at the behavioural level, i.e. patients experienced less pleasant events and social interactions resulting in more negative affect overall [25].

It is important to remember that ecological validity is not fully provided by an out-of-lab measurement. EMA is a subjective measurement method which faces similar problems as retrospective questionnaires such as individual interpretation of scales and questions or recency effects. In addition, highly intensive measurement could induce reactivity effects such as behavioural change which also threatens ecological validity [26].

#### Longitudinal Measurement Enables Chronological Associations

The repeated measurements of EMA contribute to a deeper understanding of temporally shifted associations even if the observational nature limits definitive conclusions regarding causal inference. Triantafillou et al. investigated the relationship between self-reported sleep quality and mood in depressed, anxious and healthy subjects [27]. The study revealed several interesting findings. First, they found similar associations in both directions, mood predicted sleep quality of the following night (β = 0.247; *p* < 0.001) and sleep quality predicted mood of the following day (β = 0.270; *p* < 0.001). Second, when taking into account individual differences answering self-report scales by applying z-score standardization, the effect of sleep on mood (β = 0.344; *p* < 0.001) was much larger than vice versa (β = 0.132; *p* < 0.001). Third, potential confounders such as self-reported stress, energy and focus levels as well as objectively measured physical activity, weather, day type (working day or day off) and weekday could not explain those effects [27]. Thus, it is very likely that sleep quality has an impact on next-day mood which has considerable clinical implications.

Thewissen et al. studied the temporal relationship of emotional experiences with paranoid episodes in a clinical sample with ten daily EMA ratings over 6 consecutive days [28]. Increased anxiety and decreased self-esteem significantly predicted the onset of a paranoid episode at the next time point. During the episode, patients exhibited higher levels of anxiety, depression, and anger as well as lower levels of self-esteem. Initial paranoia intensity, depression and anger further predicted episode length. The longitudinal design was able to sharpen earlier findings demonstrating the importance of self-esteem and negative emotions in paranoia [28].

Although EMA supports temporal modeling, it is still observational. Thus, even though potential confounders were ruled out, an effect of sleep quality on next-day mood was not tested in a controlled experimental setting. The same applies to the effect of anxiety and self-esteem on paranoid episodes.

#### Comparison with Retrospective Measures

An important aspect of the EMA field is validity research, which comprises different approaches such as comparison with retrospective self-reports. Ben-Zeev and Young compared momentary and retrospective ratings of depressive symptoms in hospitalized depressed patients and nonclinical controls [29]. Patients rated 7 of 13 symptoms more severe in retrospective compared to momentary assessments and showed no difference among the other 6 symptoms. Controls retrospectively rated 4 symptoms to be more severe, 4 symptoms to be less severe and showed no difference on the remaining 5 symptoms. Thus, while controls had both positive and negative biases, depressed patients tended to show a negative bias in some retrospective assessments [29].

Even if memory biases might be tackled with (close-to) momentary assessments, those assessments are not free from cognitive distortions [30]. This could be a plausible concern in tinnitus patients with comorbidities such as depression or hyperacusis where negative thought patterns or hypervigilance towards sounds might skew responses.

#### Treatment Effect

EMA has also been used to enhance understanding of the effect of treatment. Barge-Schaapveld and colleagues were interested in the effects of antidepressant treatment in the patients’ daily life experience [31]. Treatment responders reported spending more time on household chores and less time in passive leisure activities after treatment. They experienced more positive and less negative affect as well as more highly positive mood states during activities and an increase in sleep quality [31].

In a follow-up investigation, the authors studied the feasibility to predict treatment effects by early changes in momentary positive and negative affect [32]. A change in positive affect during the first week of treatment predicted HDRS (Hamilton Depression Rating Scale) score change, response and remission rates at the end of treatment (OR = −0.6/4.3/9.3, *p* < 0.001). Changes in negative affect predicted HDRS score change and remission with smaller effects (OR = −0.3/3.6, *p* < 0.01). Adding early change in positive affect to early change in HDRS score improved prediction accuracy of all treatment outcomes at the end of treatment which holds considerable implications for clinical decision making [32].

Another investigation focused on the ability to measure antidepressant treatment-related early side effects with EMA [33]. It was found that patients reported more side effects using EMA than to the General Practitioner (GP) within the first week, e.g. dizziness was reported by 5 times as many patients according to EMA. Side effects reported by EMA were associated with lower momentary quality of life and higher dropout risk. Another EMA finding of this study was that intraindividual fluctuations of momentary quality of life decreased greater for patients than for healthy controls during the treatment course [33].

Measuring treatment effects based on EMA is very promising, but there are some things to consider: Missing data could be NMAR (not missing at random) if patients drop out of treatment due to absent or negative treatment response [30]. Repeated assessments could lead to increased self-monitoring or self-reflection which may reduce the symptoms on its own [26]. Both effects, if valid, conflate treatment effects.

#### Phenotyping

Longitudinal self-rated data is further suitable to identify subgroups of patients based on their within- and between-day fluctuations. van Genugten et al. analysed EMA mood data from a period of seven days to identify latent groups of depressed patients based on their average mood, mood variability and emotional inertia [34]. Results revealed a four-profile model that differed in terms of average mood and variability, but not inertia. The first group (5% of patients) was characterized by the most negative and least variable mood, the second group (71%) by a moderately positive and variable mood, the third group (14%) by the most positive and moderate variable mood, and the fourth group (10%) by a moderately positive and high variability in mood. At baseline, the third group was less depressed than the other groups. The authors speculate the first group to represent the melancholic depression type with persistent negative mood and limited mood reactivity, while the second group could represent atypical depression which is a more common form with some degree of mood reactivity [34].

#### Conclusion: EMA in Mental Health Research

In summary, findings from mental health research fields were able to underline the power of EMA to enhance our understanding of mood, well-being, emotional experiences, and psychopathological symptoms by extending or refining results from traditional retrospective methods. As demonstrated, this methodology enabled the precise detection of short- and long-term patterns, captured differences within- and between subjects, could be combined with objective measures via passive sensing, was scalable to large sample sizes, revealed delayed or prolonged associations, captured the nuances of daily life, enhanced the understanding and early detection of treatment response, and distinguished unique symptom profiles among subgroups. It can be a valuable addition to the toolkit of researchers and clinicians as it reveals insights with significant implications for personalised interventions and more accurate diagnosis. Yet, this chapter also highlights important limitations such as participant burden influencing data quality and protocol compliance, selection bias, ecological validity, reactivity and cognitive distortions which could influence validity and reliability of EMA data. More EMA research findings can be obtained from reviews on suicidal thoughts [35], psychopathology [36], mood disorders [37], depression [38], well-being [39], substance use [40] and mobile crowdsensing [41].

### Chapter 2: Ecological Momentary Assessment in Tinnitus Research

This chapter focuses on the application of EMA within tinnitus research. It begins with an exploration of the methodological evolution of EMA and is followed by a comprehensive overview of existing studies and study designs.

#### Chronological Development of the Literature

**Fig. 1:**
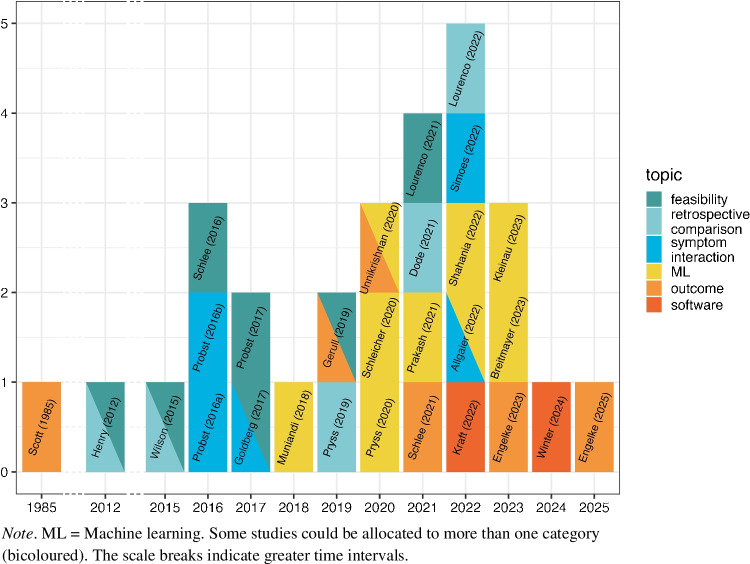
Scientific articles on EMA in tinnitus

In 1985, several years before the concept of EMA was introduced, Scott and colleagues pioneered by using daily self-recordings of tinnitus loudness, discomfort, depression and irritation to evaluate the effects of a psychological intervention [42]. Symptom severity was rated at pre-specified times on paper–pencil forms. The wider dissemination of EMA in tinnitus research began in 2012 with the publication of Henry’s pilot study investigating the feasibility of measuring within- and between-day variability of tinnitus symptoms [43]. In this work, EMA questions were thoroughly selected in a two-stage process involving focus-groups and prompts were sent out by a personal digital assistant (PDA; which can be considered the predecessor of the smartphone). Three years later, the spread of smartphones into our daily lives had enabled the authors of the second study on EMA feasibility in tinnitus to send out alerts via text messages which contained a link to the EMA survey [44]. Since then, at least one paper on EMA in the tinnitus field has been published per year (see Fig. 1). The first smartphone app that fully integrated EMA was “TrackYourTinnitus” (TYT), which collected longitudinal data on tinnitus symptoms from over 4000 patients since 2013 using a crowdsensing framework. Subsets of this data were used not only for feasibility evaluation [45], but also to enhance understanding of the longitudinal relationship and daily fluctuation of tinnitus symptoms [46, 47]. The feasibility of EMA in tinnitus has consistently been subject to research by looking at construct validity [48], compliance with dense EMA schedule [49], differences to end-of-day data (EDD) [50], and potential influence on tinnitus symptoms [51]. Further, two publications compared momentary tinnitus symptoms with measures from retrospective questionnaires [52, 53]. In 2018, the first EMA study using machine learning (ML) got published that investigated methods to capture similarities between tinnitus patients on the basis of one-time questionnaire and longitudinal EMA data [54]. Subsequently, many other ML-based analyses followed [55–62]. In recent years, the feasibility to use EMA as an instrument for collecting outcome measures in clinical studies [63–65] or to combine EMA with process mining tools to analyse temporal process patterns and further improve the understanding of tinnitus symptom variability has been explored [66]. An overview of EMA studies published over the years is depicted in Fig. 1.

#### Overview of Study Designs

The design of EMA studies in tinnitus are displayed in Table 1 (N = 28). 15 studies (54%) retrieved data from the TYT app, 6 studies (21%) sampled unique data sets, 3 studies (11%) used data from the TinnitusTipps app, 3 studies (11%) used data from the UNITI app and 2 studies (7%) used data from the TinNots app. Note that using the same app may or may not involve the use of the same data set. The studies’ sample size ranges from 3 to 3691 (Mean = 460.3, Median = 203.5). The big data sets (N > 100) used in 14 studies all came from the TYT app, with two exceptions being sampled from the UNITI app. 10 publications (36%) reported the average degree of tinnitus bother of their samples. Those who did, studied on average mild, moderate and severely bothered samples. EMAs were scheduled with a range of one to seven times daily over periods of 2 weeks to 4 months. The TYT app had no pre-defined schedule which allowed the users to fill in the assessment according to their own preferences. The repeated assessments contained between 6 and 19 questions, which can be broadly grouped into tinnitus-related questions such as distress and loudness, mental health-related questions such as mood and anxiety, somatic health-related questions such as neck tension and hearing ability, and context-related questions such as activity and location. The majority of those questions focused on the current condition, while some considered the condition of the whole day (EDD questions). Compliance to the EMA protocol was reported in 8 publications (29%) and ranged between 78.3%—91.7%, however, it was not always specified how compliance was defined or how percentage was calculated.

**Table 1:** Overview and design of EMA tinnitus studies

Dataset	N	Tinnitus bother of sample	EMA schedule	No. of quest-ions	Topics of questions	EMA/EDD	Device	Compliance	Publication	Topic of the study
Scott et al. (1985)	24	inclusion = grade 2 or 3 (occasional to severe disturbance)	2 x (5 daily × 4 weeks)*	19	t-loudness (momentary and daily), t-discomfort (momentary and daily), depression, irritation	EMA & EDD	Paper–pencil	n.a	Scott et al. (1985)	Outcome
Henry et al. (2012)	24	inclusion = moderately bothersome tinnitus	4 daily × 2 weeks	19	t-handicap, location, activity, company, t-perception, t-loudness, loudness of the environment, mood, anxiety	EMA	PDA	90%	Henry et al. (2012)	Feasibility/Retrospective comparison
Wilson et al. (2015)	20	THI median = 33, TFI median = 43	4 daily × 2 weeks	6	t-bother**, t-loudness, mood, activity, loudness of the environment, stress	EMA	Smartphone (text message with link to RedCap survey)	79.4%	Wilson et al. (2015)	Feasibility/Retrospective comparison
TrackYourTinnitus (04/2014—8/2015)	658	n.a	user-defined schedule	8	t-perception, t-loudness, t-distress, mood, arousal, stress, concentration, worst symptom	EMA	Smartphone (App)	n.a	Probst et al. (2016a) [46]	Symptom interaction
TrackYourTinnitus (xx/xxxx—02/2016)	306	n.a	user-defined schedule	8	t-perception, t-loudness, t-distress, mood, arousal, stress, concentration, worst symptom	EMA	Smartphone (App)	n.a	Probst et al. (2016b) [67]	Symptom interaction
TrackYourTinnitus (04/2014—2/2016)	857	Mini-TQ mean (sd) = 13.9 (6.0)	user-defined schedule	8	t-perception, t-loudness, t-distress, mood, arousal, stress, concentration, worst symptom	EMA	Smartphone (App)	n.a	Schlee et al. (2016)	Feasibility
Goldberg et al. (2017)	40	TFI mean (sd) = 38.2 (21.6)	2 x (4 daily × 2 weeks)*	6	t-bother**, t-loudness, mood, activity, loudness of the environment, stress	EMA	Smartphone (text message with link to RedCap survey)	88% resp. 84%****	Goldberg et al. (2017)	Feasibility/Symptom interaction
TrackYourTinnitus (xx/xxxx – 06/2016)	350	n.a	user-defined schedule	8	t-perception, t-loudness, t-distress, mood, arousal, stress, concentration, worst symptom	EMA	Smartphone (App)	n.a	Probst et al. (2017)	Symptom interaction
TrackYourTinnitus (04/2014–09/2017)	450	n.a	user-defined schedule	8	t-perception, t-loudness, t-distress, mood, arousal, stress, concentration, worst symptom	EMA	Smartphone (App)	n.a	Muniandi et al. (2018)	ML
Gerull et al. (2019)	30	inclusion = bothered by tinnitus	(7 daily × 2 weeks) + (4 daily × 8 weeks) + (7 daily × 2 weeks)	10–13	(rest, staying awake, wording)***, t-loudness, t-bother, anxiety, concentration, t-thoughts, conversation, focus, interest, worry, overwhelm	EMA & EDD***	Smartphone (text message with link to online survey)	87% (median)	Gerull et al. (2019)	Feasibility/Outcome
TrackYourTinnitus (xx/xxxx – 08/2017)	1491****	n.a	user-defined schedule	8	t-perception, t-loudness, t-distress, mood, arousal, stress, concentration, worst symptom	EMA	Smartphone (App)	n.a	Pryss et al. (2019)	Retrospective comparison
TrackYourTinnitus (xx/xxxx – 03/2020)	518	n.a	user-defined schedule	8	t-perception, t-loudness, t-distress, mood, arousal, stress, concentration, worst symptom	EMA	Smartphone (App)	n.a	Pryss et al. (2020)	ML
TrackYourTinnitus (04/2014–02/2017)	1292	n.a	user-defined schedule	8	t-perception, t-loudness, t-distress, mood, arousal, stress, concentration, worst symptom	EMA	Smartphone (App)	n.a	Schleicher et al. (2020)	ML
TrackYourTinnitus (xx/xxxx – xx/xxxx)	20	n.a	user-defined schedule	8	t-perception, t-loudness, t-distress, mood, arousal, stress, concentration, worst symptom	EMA	Smartphone (App)	n.a	Unnikrishnan et al. (2020)	ML/Outcome
TrackYourTinnitus (04/2014–03/2020)	652	Mini-TQ mean (sd) = 14.3 (5.5)	user-defined schedule	8	t-perception, t-loudness, t-distress, mood, arousal, stress, concentration, worst symptom	EMA	Smartphone (App)	n.a	Dode et al. (2021)	Retropsective comparison
TinNotes	3	TFI range = 48.8–67.6	(1 daily × 11–24 days) + (7 daily × 33 days)*	15	EDD: t-avoidance, t-annoyance, t-intrusiveness, t-interference, t-fear, t-sadness, t-pleasantness, t-distraction, t-masking, t-anger, well-being, stress, sleep quality, activity level, anxiety	EMA & EDD	Smartphone (App)	EMA: 91.7%; EDD: 83.3%	Lourenco et al. (2021)	Feasibility
TinnitusTipps	53	n.a	Multiple times per day	8	t-perception, t-loudness, t-distress, hearing ability, limitation by hearing, stress, exhaustion, hearing aid	EMA	Smartphone (App)	n.a	Prakash et al. (2021)	Feasibility
Schlee et al. (2021)	39	n.a	1 daily × 6 weeks	6	t-loudness, t-distress, mood, stress, jaw tension, neck tension	EMA	Smartphone (App)	n.a	Schlee et al. (2021)	Outcome
TrackYourTinnitus (04/2014–02/2021)	3691	n.a	user-defined schedule	8	t-perception, t-loudness, t-distress, mood, arousal, stress, concentration, worst symptom	EMA	Smartphone (App)	n.a	Allgaier et al. (2022)	ML/Symtpom interaction
TrackYourTinnitus (xx/xxxx – 12/2021)	n.a	n.a	user-defined schedule	8	t-perception, t-loudness, t-distress, mood, arousal, stress, concentration, worst symptom	EMA	Smartphone (App)	n.a	Kraft et al. (2022)	Software
TinNotes	9	TQ range = 38–81	1 daily/7 daily × 3 months	11	t-avoidance, t-annoyance, t-intrusiveness, t-fear, t-sadness, t-pleasantness, t-distraction, t-anger, well-being, stress, anxiety	EMA & EDD	Smartphone (App)	EMA: 15.8 −90.5%; EDD: 73.6—100%	Lourenco et al. (2022)	Feasibility
TinnitusTipps	21	n.a	Multiple times per day	8	t-perception, t-loudness, t-distress, hearing ability, limitation by hearing, stress, exhaustion, hearing aid	EMA	Smartphone (App)	n.a	Shahania et al. (2022)	ML
TrackYourTinnitus (xx/2014 – xx/2020)	278	Mini-TQ mean (sd) = 13.7 (5.9)	user-defined schedule	8	t-perception, t-loudness, t-distress, mood, arousal, stress, concentration, worst symptom	EMA	Smartphone (App)	n.a	Simoes et al. (2022)	Symptom interaction
TrackYourTinnitus (xx/2014 – xx/xxxx)	518	n.a	user-defined schedule	8	t-perception, t-loudness, t-distress, mood, arousal, stress, concentration, worst symptom	EMA	Smartphone (App)	n.a	Breitmayer et al. (2023)	ML
UNITI app	18	THI mean (sd) = 38.4 (15.4)	1 daily × 12 weeks	10	t-loudness, t-distress, t-loudness-max, t-distress-day, t-thoughts, emotion, jaw tension, neck tension, movement, stress	EMA & EDD	Smartphone (App)	79%	Engelke et al. (2023)	Outcome
UNITI app	476	n.a	1 daily × 12 weeks	10	t-loudness, t-distress, t-loudness-max, t-distress-day, t-thoughts, emotion, jaw tension, neck tension, movement, stress	EMA & EDD	Smartphone (App)	n.a	Kleinau et al. (2023)	ML
TrackYourTinnitus (xx/xxxx – xx/xxxx)	n.a	n.a	user-defined schedule	8	t-perception, t-loudness, t-distress, mood, arousal, stress, concentration, worst symptom	EMA	Smartphone (App)	n.a	Winter et al. (2024)	Software
UNITI app	129	THI mean (sd) = 48.6 (19.6)	1 daily × 12 weeks	10	t-loudness, t-distress, t-loudness-max, t-distress-day, t-thoughts, emotion, jaw tension, neck tension, movement, stress	EMA & EDD	Smartphone (App)	78.3%	Engelke et al. (2025)	Outcome

If dataset = publication, there is only one publication for this dataset. “xx/xxxx”: unknown start and/or end time of data collection. *PDA* Personal digital assistant. *THI* Tinnitus Handicap Inventory. *TFI* Tinnitus Functional Index. *Mini-TQ* Mini Tinnitus Questionnaire. *TQ* Tinnitus Questionnaire. *n.a.* not applicable or unknown. *There were two EMA periods. **In the last 5 min. ***3 questions were additionally asked either during the first or the last EMA survey of the day. ****Different subsets were used for data analysis

### Chapter 3: Insights from EMA Research in Tinnitus

In the following, we summarize the insights gained from EMA in tinnitus research, following the topic structure presented in Fig. 1. At the end of each section the main findings are summarized.

#### Feasibility

The perception of tinnitus is at least partly modulated by psychological processes, with attentional mechanisms playing a central role. Psychological interventions aim to facilitate habituation, enabling patients to integrate the perception of tinnitus into their sensory background [68]. A main concern about repeated questioning of tinnitus symptoms during EMA is the possibility that increased symptom awareness may exacerbate the severity of the tinnitus [43]. Yet, empirical findings could not confirm this effect. Henry et al. reported no significant differences in mean Tinnitus Handicap Inventory (THI) scores after a two-week EMA period involving four daily prompts in a clinical sample (N = 24, mean difference = 1.71, *p* = 0.50) [43]. 90% of the participants indicated increased tinnitus awareness, with most viewing this as a positive change (e.g., gaining better self-understanding). Additionally, the majority experienced no disruption to their daily routine during the study. In a cohort of regular TYT app users with at least one month of usage (N = 66), no significant differences were found between the first and last five EMA responses regarding tinnitus loudness (*p* > 0.2) and tinnitus distress (*p* > 0.7) [45]. Similarly, no differences were observed for participants using the app for less than one month (tinnitus loudness: *p* > 0.5, tinnitus distress: *p* > 0.4), thereby ruling out the possibility of a self-selection bias, i.e. patients experiencing EMA reactivity might cease app usage earlier than those who are not affected [45]. Further, Goldberg et al. demonstrated that two consecutive EMA periods had no significant effect on tinnitus distress measured by the Tinnitus Functional Index (TFI) and the Overall Global Rating of Bother Scale (GBS; N = 40) [48]. When queried, 85% of patients reported no change in their tinnitus perception, 50% noted increased awareness, 82% indicated no change in tinnitus-related distress, and 80% agreed that EMA is a valuable tool for measuring tinnitus in clinical settings. Gerull et al. applied EMA across a 12-week period (before, during, and after an intervention), and found no effects on tinnitus bother during the 2-week pre-intervention phase (N = 27) [49]. The majority of patients did not report negative side effects related to EMA. In a single-case study, Lourenco et al. evaluated the effect of EMA on tinnitus symptoms using EDD questions and standard questionnaires (N = 3) [51]. While no worsening in symptoms measured by EDD questions was observed, an improvement in general stress level was attributed to one of the three patients. No clinical changes, neither improvement nor deterioration, were found in TFI or Tinnitus Questionnaire (TQ) scores.

An interesting side finding of the feasibility studies was the high within- and between-subject variability in tinnitus distress among participants, including high fluctuations within a single day [43–45]. Wilson et al. suggested a high moment-to-moment variability with unpredictable and strong changes in tinnitus symptoms as a potential cause of tinnitus bother.

Thus, repeated assessment of tinnitus symptoms using EMA does not worsen tinnitus distress or loudness, although the latter was evaluated less frequently. While EMA may increase tinnitus awareness in clinical samples, no corresponding improvement in tinnitus distress was observed. This is a critical consideration when using EMA as an outcome measure, as any potential positive effects could confound treatment outcomes. It is important to note that these findings reflect group-level effects. Individual variability, including potential positive or negative impacts on tinnitus symptoms, cannot be entirely excluded.

#### Retrospective Comparison

When comparing the results of EMA with those of retrospective questionnaires, mixed results were found. Pryss et al. compared patients who retrospectively evaluated their tinnitus loudness as varying from day to day with patients reporting no variation and found that both groups did not differ in terms of their variation of prospectively sampled tinnitus loudness over 10 (N = 305, *p* = 0.92) and 25 days (N = 158, *p* = 0.29) [52]. They also compared the prospective association of stress with tinnitus distress and loudness among patients that retrospectively rated stress to worsen, improve and not changing their tinnitus. Again, no difference was found between groups, for all patients a higher stress-level was correlated with stronger and more distressing tinnitus (N = 675, *p* < 0.001) [52].

Dode et al. also used the TYT database to assess whether retrospective and EMA data differed in tinnitus-related distress and loudness [53]. They compared the results of the Mini Tinnitus Questionnaire (Mini-TQ; assessed before TYT usage) with the means and medians of the first 7 days of TYT usage of tinnitus distress and loudness (N = 652). The correlations range between 0.52 (1 st day) and 0.36 (5 th day) for tinnitus distress with the Mini-TQ and between 0.36 (4 th day) and 0.25 (5 th day) for tinnitus loudness with the Mini-TQ. Correlations showed a descriptive decline over the 7 days.

Henry et al. compared retrospective (THI-S) with prospective measure (EMA) of tinnitus severity. 46% of the sample obtained higher values in the THI-S, while 13% showed higher values with the EMA method [43]. A similar finding was found by Wilson et al., with a tendency for higher levels in retrospective measures such as the THI and TFI than in EMA of equivalent measures [44].

In summary, prospective assessments include relevant information about tinnitus that are hardly accessible from retrospective data, as shown by Pryss et al. [52]. Tinnitus distress measured by EMA and retrospective questionnaires show medium correlations that become smaller over time [53]. However, the comparison is limited by the timing of both assessments, the retrospective questionnaire was applied before the EMA period, thus, both assessments do not capture the same period. Last, two studies found that retrospective assessment tend to show higher values in tinnitus distress compared with momentary assessments [43, 44]. The same pattern was found in depressed patients [29]. Thus, although EMA and retrospective measures correlate to some extent, their differences show that they represent different facets of the phenomenon under study and should be treated as complementary data collection instruments.

#### Symptom Interaction

The first study to investigate tinnitus symptom interactions using EMA data was Probst et al. [46]. They were interested in the influence of tinnitus loudness on tinnitus distress and whether momentary reported stress, further divided into its component’s arousal and valence, mediated this relationship. They found a direct significant effect of tinnitus loudness on distress (N = 604, *p* < 0.001), but also a partly mediating role of stress on this effect. Further analysis revealed a partly mediating role of both components, emotional arousal and emotional valence, on the relationship between loudness and distress, with a stronger effect of valence. A final model including all three components (stress, arousal, and valence) indicated that stress and emotional valence partly mediated the loudness-distress relationship, while emotional arousal did not. A surprising result of this analysis was the magnitude of the emotional valence in the model, which was even stronger than the magnitude of stress. It should be taken into account that only data from the same assessment were analyzed [46].

A follow-up investigation of the same group investigated how tinnitus is related to the variability of emotions [67]. TYT data with at least 5 consecutive assessment points per subject were considered (N = 306). Variability in arousal was operationalized as *pulse*, while variability in valence was operationalized as *spin*. The Mini-TQ score was positively correlated with both emotion dynamics, *pulse* (*r* = 0.19; *p* < 0.05) and *spin* (*r* = 0.12; *p* < 0.05). Both *pulse* (*p* < 0.01) and *spin* (*p* = 0.01) moderated the effect of tinnitus loudness on tinnitus distress independently of each other, thus, the relationship is stronger in patients with more intense emotional variability. Neither pulse nor spin had a significant effect on the time course of tinnitus distress, but spin showed an effect on the time course of tinnitus loudness (*p* = 0.007). The higher the variability in emotional valence, the more tinnitus loudness increased over time [67].

Goldberg et al. applied multilevel modeling to describe tinnitus symptom variation between- and within-individuals over time [48]. The results showed that tinnitus bother, tinnitus loudness and general stress varied together over time within individuals. Thus, if stress increased, tinnitus bother and loudness increased as well. All measured EMA items varied together between individuals. If a patient had a higher level of stress compared to another patient, tinnitus bother, tinnitus loudness, and environmental noise were also likely to be higher, while positive feelings were likely to be lower. Probst et al. investigated if within-day variations of tinnitus symptoms followed a circadian rhythm and found that tinnitus loudness and distress were higher during the night and early in the morning compared to other times of the day, even after controlling for stress levels [47].

A more recent investigation from Simoes et al. analysed whether behavioural and emotional processes from the previous and current day influenced tinnitus symptoms using data from TYT users with data of at least 10 consecutive days (N = 278) [69]. No auto- or cross-correlation was found, i.e. tinnitus symptoms were not correlated with their level from the previous day nor with behavioural or emotional processes from the previous day on a group level. However, individualised models revealed that relationships varied massively between participants, underlining the heterogeneity of tinnitus phenotypes. While there was a positive relationship of tinnitus distress and loudness on the same day for 65% of participants, lagged relations were only found in 4%. Stress was mostly positively correlated with tinnitus distress and loudness, while other processes had both positive and negative associations with the outcome measures (e.g. concentration, emotional arousal, mood). Overall, more momentary than lagged effects were identified on an individual level. A more in-depth analysis with datasets of at least 60 consecutive days only (N = 32) revealed two subgroups, with one group demonstrating both momentary and lagged relations and the other group consisting of mostly momentary relations.

Taken together, tinnitus distress is related to tinnitus loudness, which can be partially explained by momentary stress, emotional valence, and emotional arousal, but other components might also play a mediating role [46]. The positive relationship between tinnitus distress, tinnitus loudness and stress was also shown over time within and across individuals [48]. Circadian investigations revealed higher tinnitus distress and loudness during night and early mornings, however, follow-up research could use passive sensing to investigate the extent to which ambient noise levels explain this effect. It is possible that tinnitus perception is higher during the night and early morning due to the relatively quiet environment at this time of day. More severely affected tinnitus patients had higher emotional variability in terms of valence and arousal and a stronger relationship between tinnitus distress and loudness. A high variability in emotional valence was further associated with an unfavorable time course, i.e. increase in tinnitus loudness [67]. However, individualised models revealed the heterogeneity of momentary and lagged effects between tinnitus and associated symptoms. A subgroup analysis identified two groups, one with both momentary and lagged effects and one with mostly momentary effects [69]. Thus, the condition of one day is independent of the condition of the next day for most tinnitus patients.

#### Machine Learning

The crowdsensing approach employed in the TYT study generated a significant volume of EMA data, which attracted researchers interested in leveraging machine learning algorithms to address key research questions in their field. Muniandi et al. examined the overlap between patients’ static registration data and their dynamic EMA data, based on a sample of 450 participants, and found that static data predicted dynamic similarities, though they note the preliminary nature of their results [54]. Pryss et al. studied 518 TYT users to investigate systematic differences between iOS and Android answers [55]. Different machine learning methods were able to predict the operating system with an accuracy of up to 79%, thus, it might act as a confounding factor in EMA research. Another study focused on predicting user adherence by analyzing interaction duration and continuity among 1,292 participants, revealing several notable findings [56]: First, there was a steady decline in app usage over time (N_min1 day_ = 1292, N_min2 days_ = 729, N_min3 days_ = 550, N_min10 days_ = 280). Second, stress and tinnitus perception during the first days of usage were able to predict patient return after cessation. Third, female participants showed a higher level of adherence. The same group examined the effect of non-personalised tips on the adherence towards EMA in a follow-up study (N = 20) [57]. Results showed that introducing tips later in the study, rather than from the outset, increased long-term app engagement. In another study, Prakash et al. predicted future EMA data using neighbour-based models, but noted that predictions for more distant future data were less reliable (N = 53) [58]. Their tool also effectively identified outliers in EMA behavior. Similar, Shahania et al. applied a flexible approach that compared the prediction accuracy of a global model taking all users into account with a local model that learns for each individual separately to predict the next EMA entry (N = 21) [59]. They found that local models generally outperformed global models, except in cases of sparse time-series data where global models excelled. Allgaier et al. investigated variations in tinnitus perception according to country and season [62]. Interestingly, the perception of tinnitus is more likely in summer and at higher temperatures than in winter, with country-specific patterns. The frequency of tinnitus perception also generally varies between countries. A more recent study using data from the UNITI app demonstrated the advantage of personalised models that also incorporated neighbour data over exclusively idiographic models (N = 476) [60]. However, the differences were marginal and underscored the need for better criteria to define neighbourhood relationships. Finally, Breitmayer et al. used TYT data from 518 participants to predict the presence of tinnitus based on tinnitus-unspecific dimensions such as mood, stress, arousal, and concentration, achieving a prediction accuracy of up to 78% [61].

Thus, the TYT crowdsensing platform yielded substantial insights through the application of machine learning to EMA data, whereby limitations have also been identified. Studies have shown that static questionnaire data provide information about dynamic EMA patterns, operating systems may act as confounding factors, and user adherence can be predicted by early interactions. Crowdsensing data revealed seasonal and country-specific patterns of tinnitus perception. Neighbour-based and personalised models generally improve predictive accuracy of future EMA entries, though refining criteria for neighbourhood relationships is needed.

#### Outcome Research

The first application of daily recordings in tinnitus research was, to our knowledge, realised by Scott et al. [42]. They compared the effect of a psychological treatment against a waiting-list control group on tinnitus loudness, tinnitus discomfort, depression and irritation which was measured daily over four weeks before and after treatment. Their rationale for this unusual approach, at least at that time, was to capture variations in symptoms over time as well as over different situations. By pooling the results from momentary and end-of-day questions of both periods they were able to show that the treatment group improved on those outcomes compared to the waiting-list control group. More recently, EMA has been re-discovered as outcome measure in clinical studies. Unnikrishnan et al. reported a change in distribution of EMA measured tinnitus loudness and distress from before to after the introduction of non-personalised tinnitus tips without specifying the direction of change [57]. However, the effect on tinnitus distress could not be supported by questionnaire data, as there was descriptively no change in THI values during the same time period. An observational study evaluated the effect of an auricular acupressure device on tinnitus symptoms with longitudinal EMA data [63]. Results revealed heterogenous trends in tinnitus symptoms between patients. Overall, the strongest negative trends, i.e. improvement of symptoms, were found for tinnitus loudness and general stress. When comparing the trends with a retrospectively matched control group, the treatment group showed stronger reductions in tinnitus loudness and general stress, but not in tinnitus distress. A single-arm pilot study evaluated the effect of the UNITI app with questionnaire and EMA data [64]. There was a significant improvement in tinnitus distress measured with the THI from baseline to final visit. However, when comparing EMA data from a baseline period with EMA data from an end of treatment period, no significant improvement in tinnitus distress or loudness was found. Yet, when considering EMA data from the whole intervention phase, there was a small, but significant negative trend in tinnitus distress. Further, correlation between tinnitus distress and loudness descriptively weakened over the course of the study. Most importantly, questionnaire and EMA outcomes correlated (*r* = −0.75, *p* < 0.01; *r* = 0.86, *p* < 0.001). A more recent investigation predicted clinical improvement from EMA data measured during a 12-week treatment [65]. It was found that patients who benefited from treatment had linearly declining trends in tinnitus-related thoughts, jaw tension, tinnitus loudness, a linear increase in happiness, as well as variability changes in tinnitus loudness and distress. This finding provides empirical support for EMA’s sensitivity in detecting treatment responses and offers insights into specific symptom changes occurring throughout the course of treatment [65].

#### Software

The TYT app not only measures subjective symptom data, but also tracks the context longitudinally via smartphone sensors. However, variations in smartphone software and hardware hamper a uniform interpretation of sensor data. Kraft et al. developed a method to identify and eliminate those systematic errors by means of a Principal Component Analysis and information from the EMA data [70]. Their algorithm was able to correctly detect every third faulty sensor and wrongly eliminated every seventh sensor which was in fact not biased. Winter et al. suggested the application of process mining to EMA tinnitus data in order to analyse temporal patterns and state transitions in patient-reported outcomes [66]. Process mining enables the visualization of dynamic symptom trajectories, revealing detailed process maps that capture fluctuations in tinnitus perception. By complementing traditional data-driven techniques like machine learning, it provides deeper insights into symptom variability and the influence of contextual factors. This approach may be used to enhance personalised healthcare by facilitating real-time process optimization, identifying treatment bottlenecks, and enabling targeted interventions for complex, subjective health data [66].

## Discussion

Tinnitus is a complex and heterogeneous condition which is assumed to interact reciprocally with psychological states, somatic conditions, environmental factors and social context [71–73]. Under these circumstances, EMA could represent an indispensable methodological tool in tinnitus research by providing real-time insights into the daily perception and behaviour of tinnitus patients. Although an increasing number of studies have been published over the last 10 years, compared to adjacent fields, EMA in tinnitus research is still in its infancy. The extensive use of EMA in areas such as mental health, mood disorders, and well-being provides valuable insights into the potential for EMA research in tinnitus.

We would like to point out the nature of literature reviews, such as the present one, which run the risk of being biased by omitting important parts of the literature [74]. However, we are not aware of any publications being omitted.

### Learning from Other Fields

The association of momentary tinnitus symptoms with stress and other emotional processes has been intensively studied [46, 48, 67, 69]. Except for day time [47], the relation of tinnitus’ within day-variability with behaviour or context has not been under investigation. In other words, we do not know if momentary tinnitus “spikes” are related to working hours, social interaction, (sound) environment, sleep quality, or physical activity. Yet, such information could be obtained via sensor data such as location, step count, background noise level or smartphone usage and combined with EMA to validate subjective data or control for potential confounders [22]. To our knowledge, this has not been explored in tinnitus research. It could help to understand patients’ daily life and provide critical insights into environmental factors influencing tinnitus perception and coping. For instance, background noise level is quite important for tinnitus patients, as it masks the tinnitus perception and assists patients to fall asleep, however, permanent covering could prevent habituation to tinnitus on the other hand. Thus, sensing noise level by smartphone sensors as well as tracking the listening time of music could also have relevant clinical implications. The methodological work of Kraft et al. draws attention to the potential susceptibility of sensors to systematic errors which should be taken into account [70].

Another vital lesson from EMA studies in mental health is the ability to examine delayed or bidirectional associations over time. To date, very little use has been made of this possibility of using longitudinal data in tinnitus research [69]. Sleep interference is one of the major complaints reported by tinnitus patients. Just as studies have identified the mutual influence of mood and sleep in mental health [27], similar analyses in tinnitus could clarify bidirectional associations and whether managing sleep disturbances might yield improvements in tinnitus perception or distress levels.

Furthermore, EMA’s capacity to detect subtle treatment and side effects early on has shown promise in fields such as depression [32, 33], and a similar approach could enhance tinnitus intervention research. There has already been research into how treatment response is reflected in changes in momentary tinnitus symptoms [65]. This finding could be built on to identify early indicators of therapeutic success or side effects, allowing for more dynamic and personalised treatment adjustments.

The heterogeneity of tinnitus is a major challenge in tinnitus research and has been frequently identified [9, 58, 69]. Phenotyping by means of EMA data, as achieved in mood disorder research [34], suggests that tinnitus patients could be grouped based on patterns in symptom variability and intensity. This would allow a more individualised understanding of tinnitus, distinguishing between subtypes, such as those with more predictable patterns versus highly fluctuating symptom severity. These subgroups could be further investigated and understood using demographic or clinical information. For instance, Wilson et al. hypothesized a positive relationship between moment-to-moment tinnitus variability and overall tinnitus severity [44]. Identifying different tinnitus phenotypes could be critical for research on pathophysiological mechanisms and personalised interventions.

### Critical Aspects about EMA

Despite its many advantages, EMA research also has weaknesses that need to be addressed. For a complete, comprehensive discussion we refer to [30]. A major problem is that EMA data often lack psychometric evaluation which could result from difficulties in transferring evaluation standards from cross-sectional to repeated longitudinal data [75]. As an example, reliability measures need to consider the moment-to-moment changes within subjects and distinguish those from measurement errors [30]. Another serious source of bias is that repeated assessments result in missing data patterns which are seldom completely missing at random but are often systematically missing (e.g. subjects are more likely to respond in their free time than during working hours, more distressed patients drop out earlier). There are several strategies for imputing missing data but researchers should also strain to avoid missing data in the first place [30]. This could be achieved by providing in-depth training on enrolment, gamification features, feedback on symptom patterns, or financial incentives which could improve the quantity and quality of data [76].

## Data Availability

Not applicable.

## Declarations

### Declaration of AI Use

ChatGPT 4o has been used for AI-assisted copy editing.
